# Effects of grip force on median nerve deformation at different wrist angles

**DOI:** 10.7717/peerj.2510

**Published:** 2016-09-22

**Authors:** Ping Yeap Loh, Hiroki Nakashima, Satoshi Muraki

**Affiliations:** 1 Department of Human Science, Graduate School of Design, Kyushu University, Fukuoka, Japan; 2 Research Fellow of Japan Society for the Promotion of Science, Japan; 3 Department of Human Science, Faculty of Design, Kyushu University, Fukuoka, Japan

**Keywords:** Clenched fist, Unclenched fist, Median nerve cross-sectional area, Median nerve diameter, Grip strength

## Abstract

The present study investigated the effects of grip on changes in the median nerve cross-sectional area (MNCSA) and median nerve diameter in the radial-ulnar direction (D1) and dorsal-palmar direction (D2) at three wrist angles. Twenty-nine healthy participants (19 men [mean age, 24.2 ± 1.6 years]; 10 women [mean age, 24.0 ± 1.6 years]) were recruited. The median nerve was examined at the proximal carpal tunnel region in three grip conditions, namely finger relaxation, unclenched fist, and clenched fist. Ultrasound examinations were performed in the neutral wrist position (0°), at 30°wrist flexion, and at 30°wrist extension for both wrists. The grip condition and wrist angle showed significant main effects (*p* < 0.01) on the changes in the MNCSA, D1, and D2. Furthermore, significant interactions (*p* < 0.01) were found between the grip condition and wrist angle for the MNCSA, D1, and D2. In the neutral wrist position (0°), significant reductions in the MNCSA, D1, and D2 were observed when finger relaxation changed to unclenched fist and clenched fist conditions. Clenched fist condition caused the highest deformations in the median nerve measurements (MNCSA, approximately −25%; D1, −13%; D2, −12%). The MNCSA was significantly lower at 30°wrist flexion and 30°wrist extension than in the neutral wrist position (0°) at unclenched fist and clenched fist conditions. Notably, clenched fist condition at 30°wrist flexion showed the highest reduction of the MNCSA (−29%). In addition, 30°wrist flexion resulted in a lower D1 at clenched fist condition. In contrast, 30°wrist extension resulted in a lower D2 at both unclenched fist and clenched fist conditions. Our results suggest that unclenched fist and clenched fist conditions cause reductions in the MNCSA, D1, and D2. More importantly, unclenched fist and clenched fist conditions at 30°wrist flexion and 30°wrist extension can lead to further deformation of the median nerve.

## Introduction

Carpal tunnel syndrome (CTS) is one of the most common peripheral neuropathies associated with socioeconomic burden (Palmer, Harris & Coggon, 2007) and the quality of life of CTS patients has been shown to be affected by the clinical symptoms of CTS (Atroshi et al., 1999). Workplace-related musculoskeletal disorders of the upper extremities, such as CTS, have been shown to be associated with biomechanical risk factors, such as grip force exertion during forceful hand tasks, repetitive joint movements, and wrist postures (Bao et al., 2015; Harris-Adamson et al., 2015; Violante et al., 2007; You, Smith & Rempel, 2014). Furthermore, finger movements and fingertip loading are known to cause an increase in intra-carpal tunnel pressure (Kursa et al., 2005; Kursa et al., 2006; Smith, Sonstegard & Anderson Jr, 1977). Therefore, repetitive forceful finger activities may increase the risk of CTS.

Furthermore, the non-neutral wrist posture has been shown to be associated with an overall high risk of CTS (You, Smith & Rempel, 2014). Flexed and extended wrist posture can lead to changes in the shapes of the carpal tunnel, as well as the displacement of the median nerve, finger flexor tendons and stiffness of the transverse carpal ligament (Bower, Stanisz & Keir, 2006; Holmes et al., 2011). MRI studies have suggested that wrist flexion/extension can decrease the volume of the carpal tunnel when compared to neutral wrist postures (Mogk & Keir, 2008; Mogk & Keir, 2009). Furthermore, wrist flexion and extension movements cause three-dimensional displacement of the median nerve and finger flexor tendons, namely proximal-distal, radial-ulnar, and dorsal-palmar displacements (Canuto et al., 2006; Wang et al., 2014; Yoshii et al., 2008; Yoshii et al., 2013). Subsequently, changes in wrist posture and finger movements can also influence finger flexor tendons geometry within the carpal tunnel (Keir & Wells, 1999; Martin et al., 2013). In response to the contact pressure arising from finger flexor tendon displacement, the median nerve deforms in order to adapt to the biomechanical stress (Wang et al., 2014). Deformations of the cross-sectional area and diameter of the median nerve have been reported with changes in wrist posture and finger movement via ultrasound studies (Loh, Nakashima & Muraki, 2015; Loh & Muraki, 2015; Wang et al., 2014; Yoshii et al., 2013).

The median nerve is located beneath the transverse carpal ligament and is exposed to mechanical stresses such as contact pressure from the flexor digitorum superficialis (FDS) and flexor digitorum profundus (FDP), and external compression pressure. Previous studies described the geometry changes of the finger flexor tendons with both wrist and finger movements as well as deformation of the median nerve under stress loading using ultrasound imaging (Ugbolue et al., 2005; Van Doesburg et al., 2010; Yoshii, Ishii & Sakai, 2013). The median nerve parameters such as cross-sectional area becomes smaller at both full flexion of four fingers and single finger flexion (Van Doesburg et al., 2012). For instance, independent middle finger flexion results in the FDS tendon displaced palmarly towards the transverse carpal ligament and creates contact stress on the median nerve (Yoshii et al., 2009). In addition, maximal finger flexion and forceful grip could increase the finger flexor tendon load (Keir et al., 1997) and lead to incursion of the lumbrical muscles into the carpal tunnel (Cartwright et al., 2014; Cobb et al., 1994; Cobb, An & Cooney, 1995), which can cause the cross-sectional area of the median nerve to become smaller when compared to the area with only wrist and/or finger movements.

**Figure 1:**
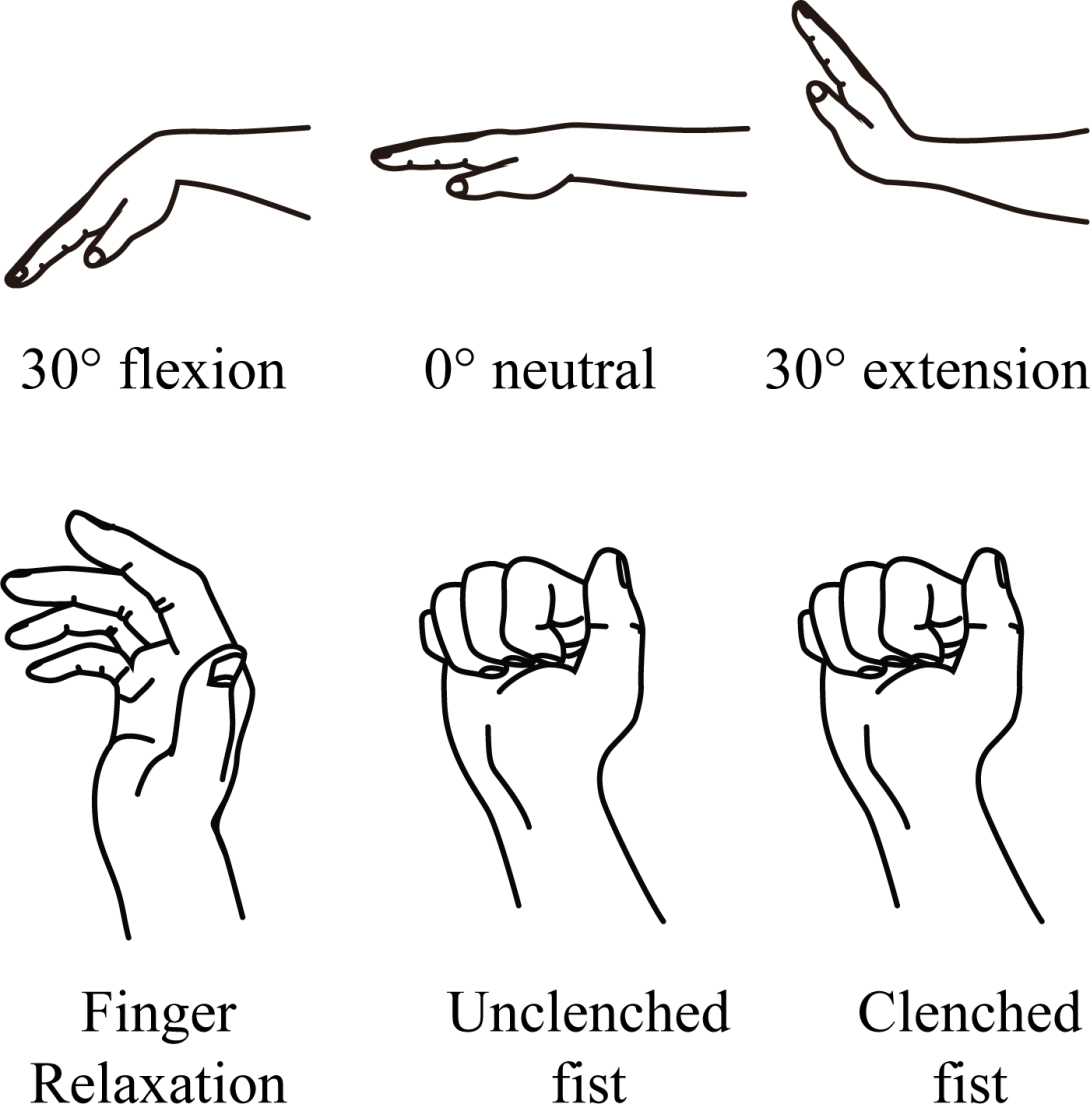
Grip conditions and wrist angles for ultrasound examination. Finger relaxation, the fingers are rest in natural curvature; Unclenched fist, full fingers flexion and hold with minimal effort; Clenched fist, full fingers flexion and exert force to grip.

To the best of our knowledge, most studies have not yet attempted to identify the impact of power grip or forceful clenched fist on changes to median nerve deformation. The clenched fist posture or forceful finger flexion posture could lead to a higher deformation of the median nerve compared to individual finger flexion or unclenched fist conditions. In addition, the deformation of the median nerve at unclenched and clenched fist conditions at different wrist angles may demonstrate a different trend due to the changes of carpal tunnel size. The primary objective of the present study was to investigate the impact of three types of grip conditions (finger relaxation, unclenched fist, and clenched fist) (Fig. 1) on changes in the median nerve cross-sectional area (MNCSA) and median nerve diameter in the radial-ulnar direction (D1) and dorsal-palmar direction (D2) at three wrist angles. We hypothesized that the MNCSA and median nerve diameter will become smaller with an unclenched and clenched fist, relative to finger relaxation at a neutral wrist position. Secondly, wrist extension and flexion will cause the MNCSA and median nerve diameter to become smaller, compared to the neutral wrist condition. Next, we postulated that grip strength of the dominant hand would be stronger than the nondominant hand at three wrist angles.

## Materials and Methods

### Participants

Twenty-nine healthy young adults (Table 1) without known upper limb musculoskeletal disorders were recruited. The handedness of the participants was determined with the Edinburgh Handedness Inventory (Oldfield, 1971). The participants provided written informed consent, and this study was approved by the Ethics Committee of the Faculty of Design, Kyushu University (Approval number 141).

**Table 1:** Characteristics of the participants (*n* = 29).

		Male (*n* = 19)	Female (*n* = 10)
Age (years)		24.2 ± 1.6	24.0 ± 1.6
Height (cm)		171.5 ± 4.7	159.1 ± 4.7
Weight (kg)		61.7 ± 6.0	48.9 ± 6.4
BMI (kg/m^2^)		21.0 ± 2.1	19.3 ± 2.1
Handedness (Right : left hand dominant)		18 : 1	8 : 2
Grip strength (kgf)			
30° wrist flexion	Dominant hand	23.6 ± 5.8	11.8 ± 2.1
	Nondominant hand	20.1 ± 4.6	12.3 ± 2.4
Neutral wrist (0°)	Dominant hand	28.9 ± 6.8	15.8 ± 2.9
	Nondominant hand	25.6 ± 6.2	14.9 ± 3.3
30° wrist extension	Dominant hand	33.8 ± 7.5	19.2 ± 4.5
	Nondominant hand	29.9 ± 6.9	17.5 ± 4.2

### Grip strength assessment

The grip strength of the participants was assessed using the digital grip strength dynamometer Grip-D (T.K.K. 5401; Takei Scientific Instruments Co., Ltd., Niigata, Japan). With the intention of simulating clenched fist with full interphalangeal joint flexion, the grab bar of the dynamometer was positioned at level 4 during grip strength assessment (Fig. S1). The participants positioned the forearm in mid-pronation on an arm support during the grip assessment. A wrist goniometer (Exacta™, North Coast Medical Inc., Morgan Hill, CA, USA) was used to determine the wrist angle. The axis point of the goniometer was placed at the triquetrum, while static and movable bars were placed parallel to the ulna bone and 5th metacarpal bone, respectively. The goniometer was used to determine the wrist angle before the grip strength assessment and to ensure that the wrist was maintained at the designated angle during the grip strength assessment. The grip strength of both the dominant and nondominant hands were measured thrice at three wrist positions (wrist flexion (30°), neutral position (0°), and wrist extension (30°)). The mean of the three grip strength measurements was calculated for each wrist position (Table 1).

### Ultrasound examination protocol

The LOGIQ e ultrasound system (GE Healthcare, Milwaukee, WI, USA) with a 12L-RS transducer (imaging frequency bandwidth of 5–13 MHz) was used to examine the wrist. A 7.0-mm-thick sonar pad (Nippon BXI Inc., Tokyo, Japan) was used as a coupling agent during the ultrasound examination. The examiner placed the ultrasound transducer gently on the sonar pad to avoid compression pressure at the wrist during the examination. The forearm was positioned in supination and rested on an arm support on a table, with the elbow at 30° flexion, during the ultrasound examination. The examiner placed the ultrasound transducer parallel to distal wrist crease to identify the median nerve in the transverse plane, with the pisiform as the anatomical landmark in all conditions. A custom made L-shape frame was used to assist the examiner to place the transducer perpendicularly to the wrist. Similar to the approach in the grip strength assessment, a wrist goniometer was placed at the ulnar side of the wrist and was used to position the wrist at the designated angle for each image. In addition, the ultrasound transducer was removed and repositioned, and wrist angle was re-measured before each image was obtained. The following three grip conditions were examined: finger relaxation (control condition), unclenched fist, and clenched fist (Fig. 1). Three images were taken for each posture at 30° wrist flexion, in the neutral position (0°), and at 30° wrist extension for both the dominant and nondominant hands.

### Image processing and analysis

**Figure 2:**
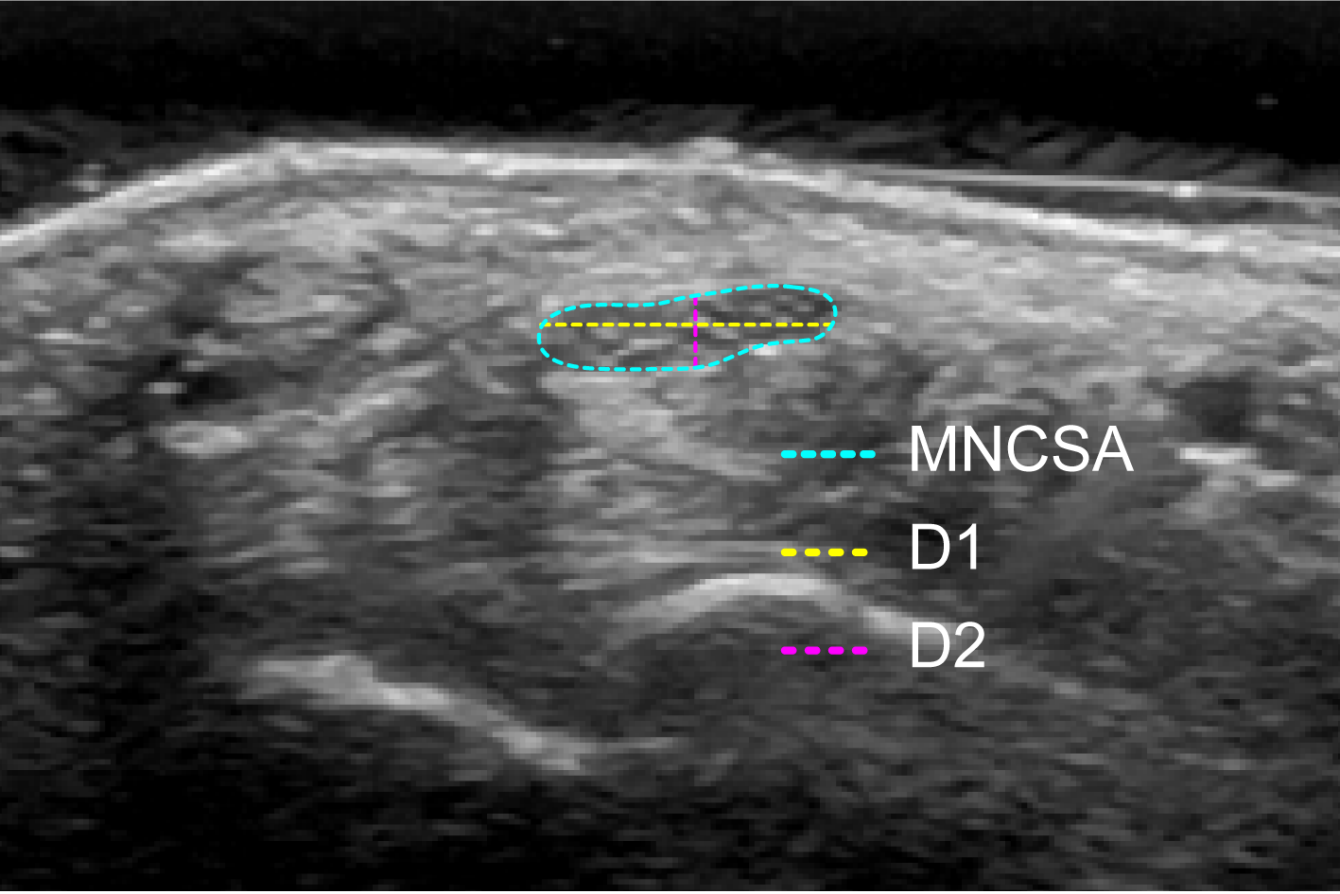
Quantification of the median nerve cross-sectional area (MNCSA), diameter in radial-ulnar direction (D1) and diameter in dorsal-palmar direction (D2).

The MNCSA, D1, and D2 (Fig. 2) were quantified using ImageJ software (National Institutes of Health) (Schneider, Rasband & Eliceiri, 2012). The median nerve was identified as a hyperechoic structure in the transverse plane (Kele, 2012), and then, the MNCSA was quantified with the tracing method (Duncan, Sullivan & Lomas, 1999). Subsequently, the examiner traced the median nerve along the hyperechogenic rim, and then, the longest diameter in the radial-ulnar direction (D1) and dorsal-palmar direction (D2) were identified by two perpendicular straight lines within the outlined median nerve (Fig. 2). This quantifying method was found to have good to excellent inter- and intra-rater reliabilities in a previous study (Loh & Muraki, 2015). The mean of three images was calculated for the MNCSA, D1, and D2 at each finger posture. The deformation percentages of the MNCSA, D1, and D2 were calculated using the following equation:

\begin{eqnarray*}\begin{array}{@{}l@{}} \displaystyle \text{Deformation percentage}=\\ \displaystyle ~ \frac{\text{Measurement at a different grip condition}-\text{Measurement at finger relaxation at neutral wrist}}{\text{Measurement at finger relaxation at neutral wrist}} \times 100\text{%} \end{array} \end{eqnarray*}

### Statistical analysis

Two-way repeated analysis of variance (3 × 2 factorial design) was performed with three wrist angles (30° flexion, neutral (0°), and 30° extension) and hand dominance (dominant and nondominant) as factors to examine differences in the grip strength.

The sample characteristics of the MNCSA was examined with the Shapiro–Wilk’s normality test (Razali & Wah, 2011; Shapiro & Wilk, 1965). Two-way repeated analysis of variance (3 × 3 factorial design) was performed with three grip conditions (finger relaxation, unclenched fist, and clenched fist), and three wrist angles (30° flexion, neutral (0°), and 30° extension) as factors to examine differences in MNCSA, D1, and D2. Post-hoc pairwise Bonferroni-corrected comparison was performed to examine the significant effects. Significance was set at *α* = 0.05. All statistical analyses were performed using SPSS version 21.0 software (IBM Corp., Armonk, NY, USA). The results are presented in mean ± standard deviation.

## Results

### Grip strength at different wrist angle

The main effect of wrist angle on the change of grip strength was significant (*p* < 0.01). The grip strength at neutral wrist was significantly stronger (*p* < 0.05) than that at 30° wrist flexion but significantly weaker (*p* < 0.01) than that at 30° wrist extension. However, the main effect of hand dominance was not significant.

### Sample characteristics

The MNCSAs were approximately normally distributed in the Shapiro–Wilk’s test (*p* > 0.05), and the samples were slightly skewed and kurtotic on visual inspection of histograms, normal Q–Q plots, and box plots (Table 2).

**Table 2:** Normality test for the median nerve cross-sectional area.

Skewness (M ± SE)	Kurtosis (M ± SE)	Shapiro–Wilk test (*p* value)
0.44 ± 0.31	0.23 ± 0.62	0.490

**Notes.**

M mean SE standard error

**Figure 3:**
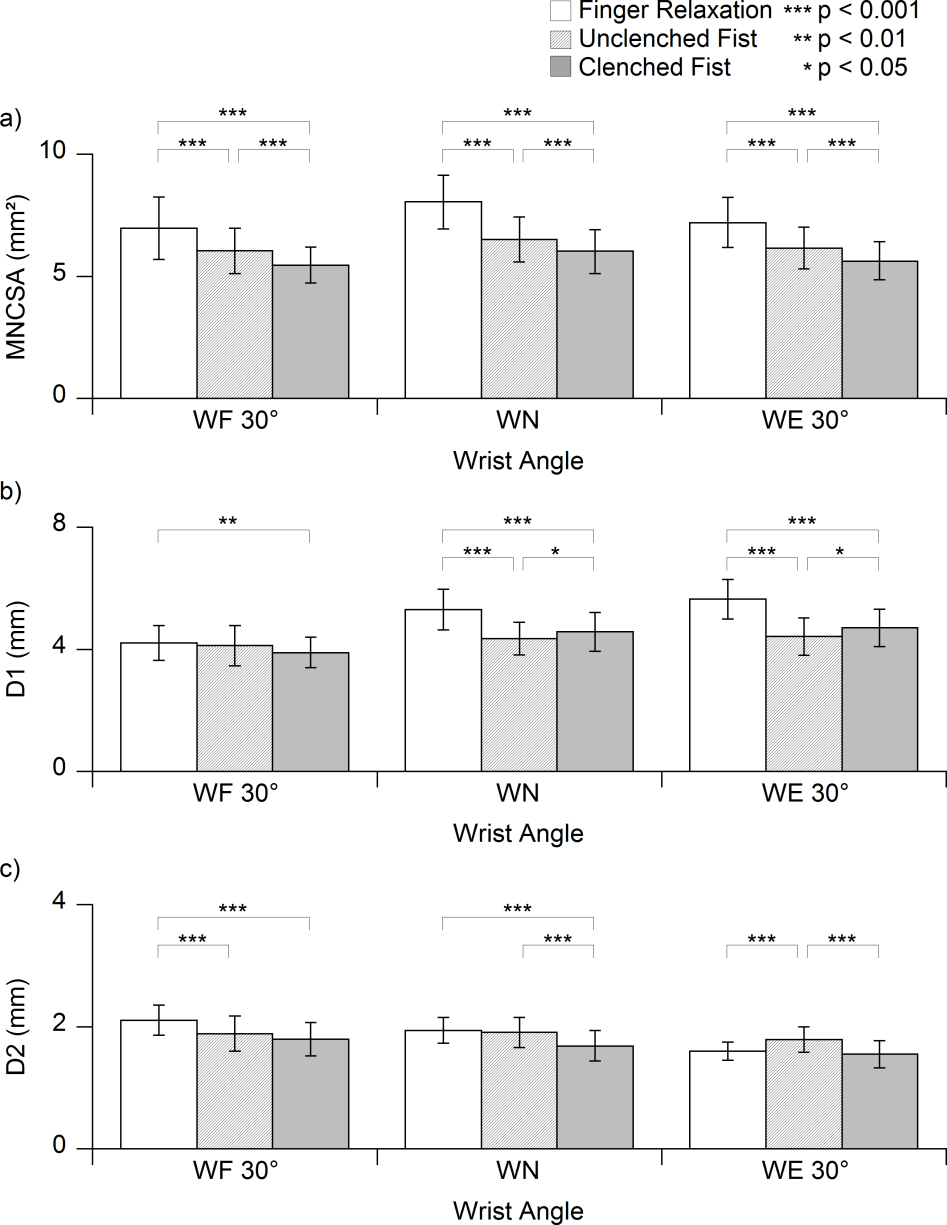
Mean value of the (A) median nerve cross-sectional area (MNCSA) (B) longitudinal diameter (D1) and (C) vertical diameter (D2) of finger relaxation, unclenched fist and clenched fist at three wrist angles. WF, wrist flexion; WN, wrist neutral; WE, wrist extension; MNCSA, median nerve cross-sectional area; D1, diameter in radial-ulnar direction; D2, diameter in dorsal-palmar direction.

### Effect of grip conditions and the wrist angle on the change in the MNCSA

The main effects of the grip condition (*p* < 0.001) and wrist angle (*p* < 0.001) on the change in the MNCSA were significant. Furthermore, a significant interaction was found between the grip condition and wrist angle (*p* < 0.01). The MNCSA significantly reduced as finger relaxation changed to unclenched fist and clenched fist conditions at all three wrist angles (Fig. 3A). The MNCSA at clenched fist was the smallest among the three grip conditions. The MNCSA was significantly smaller at wrist flexion (30°) and extension (30°) than in the neutral position (0°) in each grip condition (Table 3A). The deformations caused by unclenched fist and clenched fist conditions were approximately −20% and −30%, respectively (Table 4A) for the three wrist angles.

**Table 3:** Median nerve cross-sectional area and diameters of each grip condition at different wrist angles.

Wrist angle	Grip conditions		
	Finger relaxation	Unclenched fist	Clenched fist
**(A) Median nerve cross-sectional area (MNCSA, mm^2^)**			
30° Flexion	7.0 ± 1.0^a^	6.0 ± 0.9^a^	5.5 ± 0.7^a^
0° Neutral	8.1 ± 1.3	6.5 ± 0.9	6.0 ± 0.9
30° Extension	7.2 ± 1.1^a^	6.2 ± 0.9^a^	5.6 ± 0.8^a^
**(B) Diameter in radial-ulnar direction (D1, mm)**			
30° Flexion	4.2 ± 0.6^a^	4.1 ± 0.7 ^c^	3.9 ± 0.5^b^
0° Neutral	5.3 ± 0.7	4.4 ± 0.5	4.6 ± 0.6
30° Extension	5.6 ± 0.7^b^	4.4 ± 0.6	4.7 ± 0.6
**(C) Diameter in dorsal-palmar direction (D2, mm)**			
30° Flexion	2.1 ± 0.2^c^	1.9 ± 0.3	1.8 ± 0.3^c^
0° Neutral	1.9 ± 0.2	1.9 ± 0.3	1.7 ± 0.3
30° Extension	1.6 ± 0.1^c^	1.8 ± 0.2^c^	1.6 ± 0.2^c^

**Notes.**

a *p* < 0.001.

b *p* < 0.01.

c *p* < 0.05.

post-hoc Bonferroni with compared to the wrist neutral at each grip condition.

### Effect of grip conditions and the wrist angle on the changes in D1 and D2

The main effects of the grip condition (D1, *p* < 0.01; D2, *p* < 0.01) and wrist angle (D1, *p* < 0.01; D2, *p* < 0.01) on the changes in the median nerve diameter were significant. Furthermore, a significant interaction was found between the grip condition and wrist angle for both D1 and D2 (D1, *p* < 0.001; D2, *p* < 0.001). Generally, D1 significantly reduced as finger relaxation changed to unclenched fist or clenched fist conditions (Fig. 3B). Similarly, D2 significantly reduced as finger relaxation changed to unclenched fist or clenched fist conditions, except at 30° wrist extension (Fig. 3C). At 30° wrist extension, D2 was significantly higher at unclenched fist condition than at finger relaxation and clenched fist condition(Fig. 3C).

**Table 4:** Deformation percentage (%) of the median nerve cross-sectional area and diameters of each grip conditions at different wrist angles.

Wrist angle	Grip conditions		
	Finger relaxation	Unclenched fist	Clenched fist
**(A) Median nerve cross-sectional area (MNCSA, mm^2^)**			
30° Flexion	−12.8	−18.7	−31.7
0° Neutral	NA	−22.9	−24.5
30° Extension	−10.1	−22.9	−29.5
**(B) Diameter in radial-ulnar direction (D1, mm)**			
30° Flexion	−20.3	−21.9	−25.7
0° Neutral	NA	−17.3	−12.8
30° Extension	6.7	−16.2	−10.4
**(C) Diameter in dorsal-palmar direction (D2, mm)**			
30° Flexion	9.3	−2.1	−6.8
0° Neutral	NA	−0.9	−12.5
30° Extension	−17.0	−6.9	−19.4

Additionally, the wrist angle changes caused significant deformation of D1 and D2 in each grip condition. D1 reduced as the wrist angle changed from 30° extension to neutral (0°) and from neutral (0°) to 30° flexion (Table 3B). In contrast, D2 increased as the wrist angle changed from 30° extension to neutral (0°) and from neutral (0°) to 30° flexion (Table 3C). In general, the highest deformation percentage of D1 was at 30° flexion (approximately −25%) (Table 4B), while the highest deformation percentage of D2 was at 30° extension (approximately −19%) (Table 4C).

## Discussion

### Effects of grip conditions on the changes in the MNCSA, D1, and D2 in the neutral wrist position (0°)

Biomechanics factors for injury in workplace, such as hand force and wrist posture, are known to be risk factors for CTS among workers (Burt et al., 2013). The differential excursion amplitude of the finger flexor tendons and the force exertion of the finger flexor muscles could contribute to the changes in median nerve tension and intra-carpal tunnel pressure, and thus affect the deformation of the median nerve.

In this study, we examined the changes of the MNCSA and the median nerve diameter among finger relaxation, unclenched fist and clenched fist, conditions (Fig. 1) in the neutral wrist position (0°) by ultrasound imaging technique among healthy young adults. We found a significant reduction in the MNCSA as the fingers changed from finger relaxation to unclenched fist condition (Fig. 3A), which may have resulted from mechanical stress arising from the radial-ulnar displacement of the finger flexor tendons within the carpal tunnel (Van Doesburg et al., 2010). Subsequently, a further reduction in the MNCSA was observed as the fingers changed from unclenched fist condition to clenched fist condition (Fig. 3A). The maximal excursion and displacement of both the FDS and FDP secondary to the finger flexor muscles bellies contraction might have caused further transverse contraction stress to the median nerve within the confined carpal tunnel space. The higher deformation percentage of the MNCSA at clenched fist condition (Table 4A) indicates the cross-sectional area of median nerve becomes smaller which could have resulted from an increase in the intra-carpal tunnel pressure caused by maximal excursion of the finger flexor tendons at clenched fist condition (Goss & Agee, 2010).

We then analyzed the changes in D1 and D2 at different grip conditions in the neutral wrist position (0°). We found that D1 and D2 were significantly lower at unclenched fist and clenched fist conditions than at finger relaxation (Figs. 3B and 3C). Interestingly, clenched fist conditions did not result in the highest deformations of both D1 and D2 (Fig. 3). We found that the highest deformations of D1 and D2 were at unclenched fist and clenched fist conditions, respectively (Table 4B and 4C). This phenomenon may have been caused by contact stress from the finger flexor tendons within the carpal tunnel and the different elongation degrees of the median nerve at each grip condition. Our results indicated that active contraction of FDS and FDP during our clenched fist condition caused a greater change in median nerve diameter than the unclenched fist condition. Consequently, elongation of the median nerve secondary to clenched fist condition may affect the changes in D2 compared to to unclenched fist condition at neutral wrist position. Therefore, the finger flexion force may be one of the important factors contributing to the dynamic changes in the median nerve diameter, as observed at unclenched fist and clenched fist conditions (Figs. 3B and 3C).

### Effects of grip conditions and the wrist angle on changes in the MNCSA, D1, and D2

The space within the epineural tube of peripheral nerves is crucial for the nerve to adapt to the external mechanical stress from surrounding structures. The median nerve is displaced and slides between the finger flexor tendons, and its shape is altered in response to irregular displacement of the finger flexor tendons within the carpal tunnel. We analyzed the effects of wrist angle on changes to the median nerve in different grip conditions to address our second hypothesis.

At finger relaxation, the MNCSA was significantly lower at 30° wrist flexion and 30° wrist extension than in the neutral wrist position (0°) (Table 3A). The reductions in the MNCSA caused by wrist flexion and extension are consistent with the findings of previous studies that showed deformation of the median nerve caused by wrist movements in young and old adults (Loh & Muraki, 2015). Furthermore, the MNCSA significantly reduced as the wrist changed from neutral (0°) to 30° flexion and 30° extension (Table 3A) at both unclenched fist and clenched fist conditions (Fig. 1). Additionally, at unclenched fist and clenched fist conditions, the deformation percentages of the MNCSA were higher at 30°wrist flexion and 30° wrist extension than in the neutral wrist position (0°) (Table 4A). Previous studies have shown that the carpal tunnel volume is lower at wrist flexion and extension that in the neutral wrist position (0°) (Mogk & Keir, 2008). The gliding amplitude of the finger flexor tendons increased during the clenched fist condition, and this could lead to incursion of the lumbrical muscles into the distal carpal tunnel (Cobb et al., 1994). Gliding of the finger flexor tendons in a small carpal tunnel and incursion of lumbrical muscles into the carpal tunnel could substantially increase the intra-carpal tunnel pressure and result in high deformation of the MNCSA (Table 4A).

Our results are consistent with those of previous studies showing that wrist flexion causes significant changes in D1 at finger relaxation (Loh & Muraki, 2015). D1 was significantly lower at 30° wrist flexion and was significantly higher at 30° wrist extension than in the neutral wrist position (0°) at finger relaxation (Table 3B). Additionally, 30° wrist flexion and 30° wrist extension resulted in a further reduction in D1 at unclenched fist and clenched fist conditions (Table 3B). Furthermore, wrist flexion resulted in a further significant reduction in D1 at clenched fist condition but not at unclenched fist condition (Table 4B). Although D1 showed a decreasing trend at wrist flexion with unclenched fist condition, there was no significant difference on comparing wrist flexion with the neutral wrist position (Table 4B). Notably, the deformation of D1 in all the three grip conditions was higher at 30° wrist flexion than at the other wrist angles (Table 4B). This further reduction of D1 could possibly have resulted from the changes of geometry arrangement of the finger flexor tendons within a smaller carpal tunnel at wrist flexion and extension.

In contrast, D2 was significantly lower at finger relaxation and clenched fist condition than at unclenched fist condition at 30° wrist extension (Fig. 3C). The deformation percentages of D2 were generally lower than the deformation percentages of D1 in all grip conditions and at all wrist angles, and the largest deformation percentage was approximately −20% (Table 4C). This reduction in D2 could have resulted from elongation and the presence of transverse contraction stress at the median nerve. The stiffness of the transverse carpal ligament has been reported to be higher at wrist flexion than at wrist extension and in the neutral wrist position (Holmes et al., 2012). We found that D2 was significantly higher at unclenched fist condition than at both finger relaxation and clenched fist condition at 30° wrist extension (Fig. 3C). The low stiffness of the transverse carpal ligament at 30° wrist extension (Holmes et al., 2011) might have resulted in low dorsal-palmar stress on the median nerve at unclenched fist condition.

The present study has some limitations. First, the rotational axis of the median nerve could not be identified owing to the image acquisition protocol. The displacement of the finger flexor tendons could lead to changes in the rotational axis of the median nerve and could affect the quantification of median nerve diameter. The rotational effects of the median nerve should be considered in future studies. Second, our results indicate that hand dominance does not affect grip strength at different wrist angles. The measured grip strengths among both male and female participants in the present study (Table 1) were approximately 40–50% lower than the reported normative data (Dodds et al., 2014; Dodds et al., 2016; Massy-Westropp et al., 2011; Nagasawa, Demura & Hamazaki, 2010). The lower grip strength data obtained in this study most likely resulted from the low grab bar position of the dynamometer and the forearm posture during grip strength assessment (Fig. S1), which was different from the posture during grip strength assessment reported previously (Massy-Westropp et al., 2011). A higher grip force exertion could lead to a higher compression stress to the median nerve which results in further decrease of MNCSA. Future studies with different power grip span is needed to investigate the impact of gripping force on the median nerve deformation.

## Conclusion

Our study showed that the median nerve deforms with finger flexion movements, while clenched fist condition contributes to higher deformation percentages of median nerve parameters. Furthermore, we demonstrated that the wrist angle was an important factor that could affect the deformability of the median nerve in unclenched and clenched fist conditions. In summary, wrist flexion and extension can lead to higher deformation of the MNCSA, and wrist flexion and extension influence the deformation of both D1 and D2, respectively. Future studies are needed to further explore the impacts of grip and the kinematic changes of the finger flexor tendons on the deformation of the median nerve, with special consideration of median nerve mobility.

##  Supplemental Information

10.7717/peerj.2510/supp-1 Supplemental Information 1 Grip strength

10.7717/peerj.2510/supp-2 Figure S1 (A) Normal grip span used in grip strength assessment; (B) Adjusted grip span used in this study

## Additional Information and Declarations

### Data Availability

The following information was supplied regarding data availability: The raw data has been supplied as a Supplementary File.
