# Supplementary figures and images for: Effects of grip force on median nerve deformation at different wrist angles
Source: PeerJ. 2016 Sep 22;4:e2510. doi: 10.7717/peerj.2510 (PMC5036108; doi:10.7717/peerj.2510)

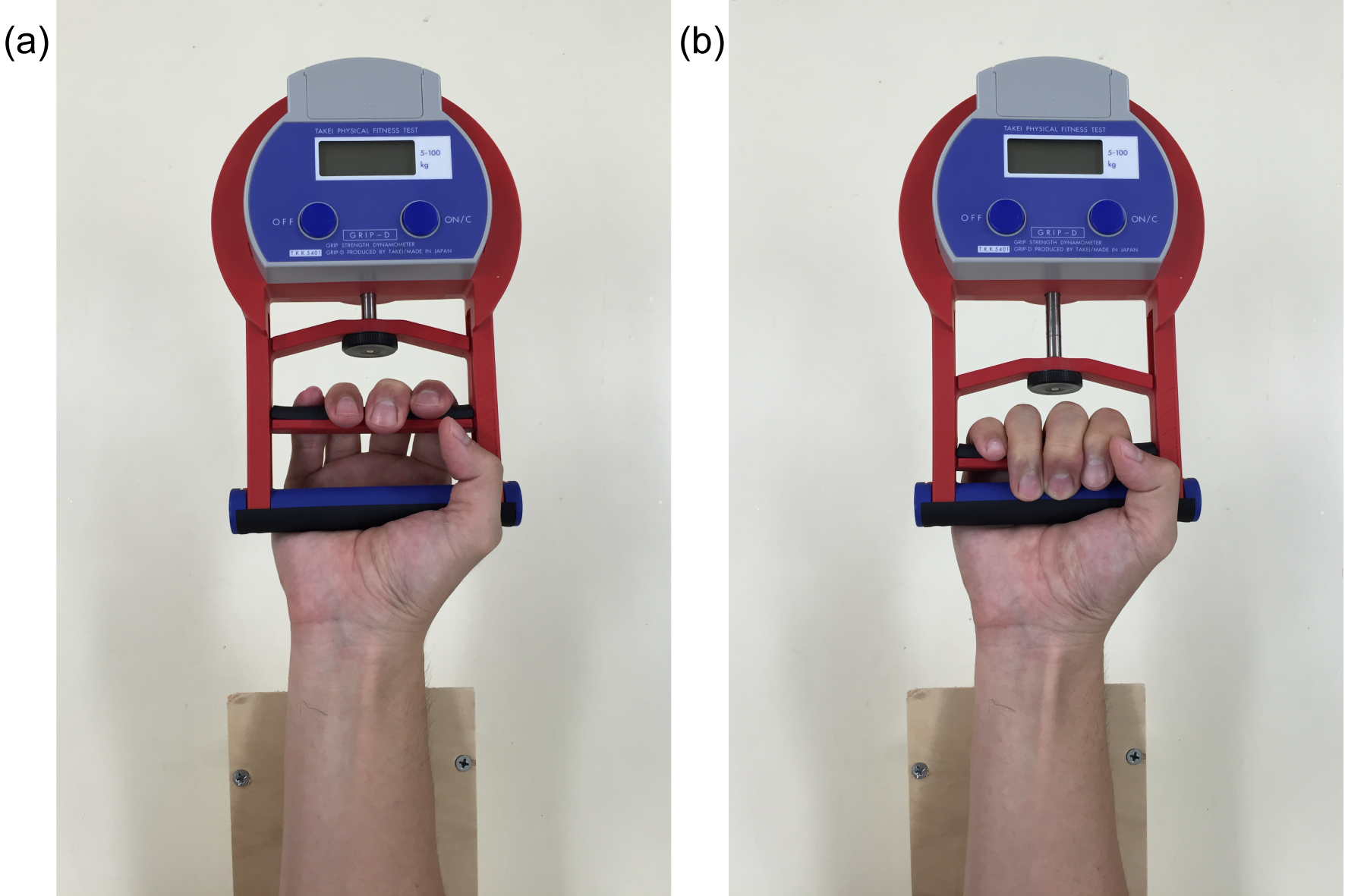

Supplement: Figure S1 [file peerj-04-2510-s002.png]
